# DNA-barcoded signal amplification for imaging mass cytometry enables sensitive and highly multiplexed tissue imaging

**DOI:** 10.1038/s41592-023-01976-y

**Published:** 2023-08-31

**Authors:** Tsuyoshi Hosogane, Ruben Casanova, Bernd Bodenmiller

**Affiliations:** 1 Department of Quantitative Biomedicine, University of Zurich, Zurich, Switzerland; 2 Institute for Molecular Health Sciences, ETH Zurich, Zurich, Switzerland; 3 Life Science Zurich Graduate School, ETH Zurich and University of Zurich, Zurich, Switzerland

**Keywords:** Molecular imaging, DNA probes, Proteomics, Mass spectrometry, Nanobiotechnology

## Abstract

Imaging mass cytometry (IMC) is a highly multiplexed, antibody-based imaging method that captures heterogeneous spatial protein expression patterns at subcellular resolution. Here we report the extension of IMC to low-abundance markers through incorporation of the DNA-based signal amplification by exchange reaction, immuno-SABER. We applied SABER-IMC to image the tumor immune microenvironment in human melanoma by simultaneous imaging of 18 markers with immuno-SABER and 20 markers without amplification. SABER-IMC enabled the identification of immune cell phenotypic markers, such as T cell co-receptors and their ligands, that are not detectable with IMC.

## Abstract (web summary)

SABER-IMC combines DNA-based signal amplification by exchange reaction (SABER) with imaging mass cytometry (IMC) to enable simultaneous and highly multiplexed marker detection, even of low-abundance markers not detectable with IMC alone.

## Main

Highly multiplexed, antibody-based imaging enables spatial investigation of protein expression within tissues^1–9^. In the tumor microenvironment (TME), multiplexed imaging has shown that multiple types of tumor, immune and stromal cells, and their interactions, drive the disease^10^. Imaging of more than five targets is challenging with standard immunofluorescence imaging due to spectral overlap of fluorophores. Higher multiplexity has been achieved with techniques that use iterative imaging cycles, but these cause tissue damage and are limited by autofluorescence and low dynamic range^4–9^. IMC achieves highly multiplexed (up to 40-plex) imaging^11^ using metal isotope-labeled antibodies to stain biological samples, followed by pixel-wise focused laser ablation and simultaneous detection of these labeled antibodies in a mass cytometer^12^. However, imaging of low-abundance proteins using IMC is challenging. Standard amplification approaches using secondary antibodies cannot be multiplexed due to the low number of orthogonal host species for primary antibodies. Therefore, to extend highly multiplexed IMC to low-abundance targets, a highly multiplexed signal amplification approach is needed.

Multiplexed amplification has been achieved for immunofluorescence imaging using the DNA-based signal amplification by exchange reaction (SABER) method^13^. Immuno-SABER amplifies the fluorescent signal using DNA-barcoded antibodies and cyclic hybridization of fluorescently labeled imager strands to the concatemer containing multiple repeats of barcode sequence; it has been previously used for 10-plex imaging of biological tissues^13^. Here, we implemented SABER-IMC by labeling imager strands with a metal isotope instead of a fluorophore, enabling the imaging of low-abundance target proteins and eliminating laborious iterative imaging cycles. We used 38-plex SABER-IMC to image human melanoma tissue, with a focus on low-abundance immunomodulatory molecules, including CTLA-4, PD-1 and LAG-3, and characterized cell phenotypes and interactions within the TME that would have gone undetected using standard IMC.

**Fig. 1:**
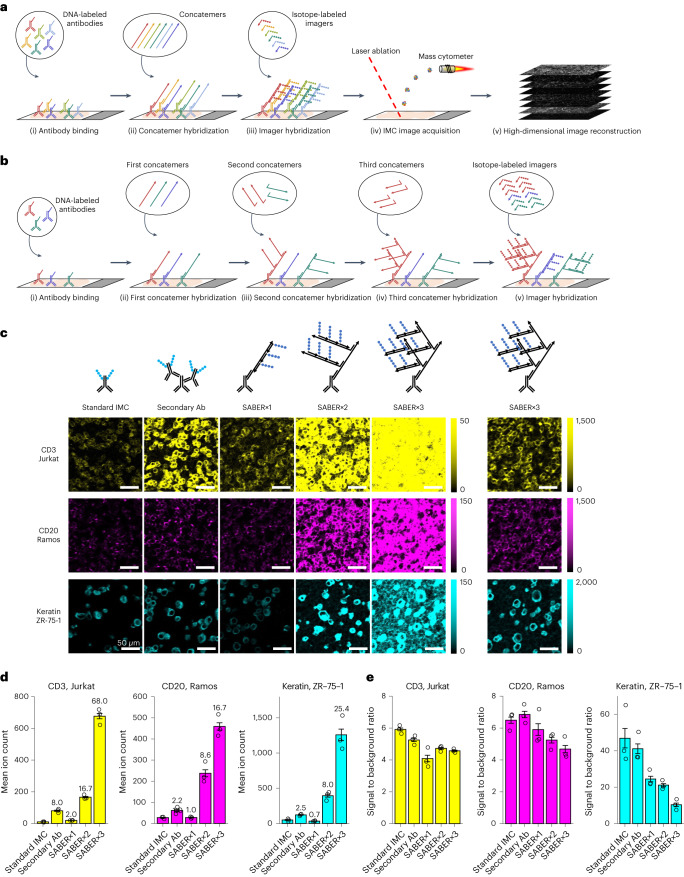
SABER-IMC workflow and quantification of signal amplification. **a**, General SABER-IMC workflow. (i) Target molecules in biological samples are recognized by antibodies labeled with unique bridge-DNA tags. (ii) Concatemers are hybridized to complementary bridge-DNA tags. (iii) Isotope-labeled imager strands are hybridized to corresponding barcode sequences on the concatemers. (iv) All isotope-labeled target molecules are simultaneously imaged by pixel-wise laser ablation and mass cytometry. (v) Mass cytometry analysis reveals isotope content of every ablation spot, enabling the reconstruction of images for each target molecule. **b**, Workflow of SABER×3-IMC for further signal amplification. (i) Target molecules are recognized by antibodies labeled with unique bridge-DNA tags. (ii)–(iv) First-round concatemers are hybridized to corresponding bridge-DNA tags, and second- and third-round concatemers are sequentially added to give additional binding sites for imagers by creating branched DNA structures. The degree of amplification can be adjusted for individual targets by selecting the number of concatemer incubation rounds. (v) Isotope-labeled imager strands are hybridized to corresponding concatemers. **c**, Images of sections of a mixed cell pellet stained with CD3 (yellow), CD20 (magenta) and keratin (cyan) with SABER-IMC with the indicated rounds of amplification, with standard IMC, or with isotope-tagged secondary antibody-based amplification. Intensity scales are indicated on the right of images. Separated column at right shows SABER×3 image with adjusted intensity scale. Scale bars, 50 µm; Ab, antibody. **d**, Bar plots of mean signal intensity of each marker in images of indicated samples. Mean signal intensity was obtained using pixels expressing higher signal than defined threshold. Fold amplification compared to standard IMC is indicated on top of each bar. Error bars are s.e.m. (*n* = 3 independent experiments; each experiment is a mean value of three regions of interest (ROIs)). **e**, Signal-to-background ratio for the indicated amplification conditions. Signal intensity and background intensity were obtained from average intensity of pixels expressing higher or lower signal than defined threshold, respectively. Error bars are s.e.m. (*n* = 3 independent experiments; each experiment is a mean value of three ROIs).

**Extended Data Fig. 1:**
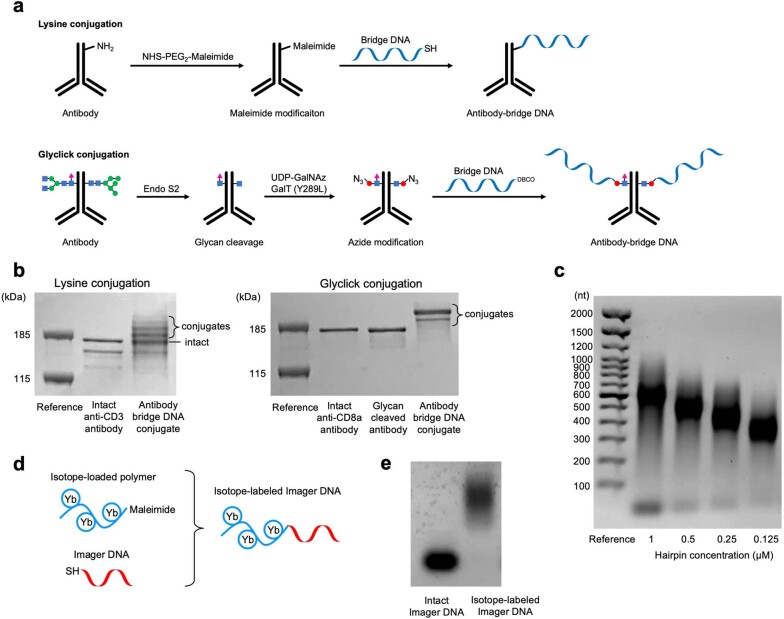
Preparation of DNA constructs for SABER-IMC. **a**, Schematic of conjugation of bridge DNA to antibodies. For lysine conjugation, random amine groups on the antibody were modified with maleimide using NHS-PEG_2_-maleimide, then conjugated with thiol-modified bridge-DNA. For Glyclick conjugation, a commercially available Glyclick kit was used for azide modification of the antibody. Briefly, glycans of the antibody were enzymatically cleaved, an azide-modified sugar was enzymatically conjugated, and then DBCO-modified bridge-DNA was conjugated via a click reaction. **b**, SDS-PAGE analysis of DNA-conjugated antibodies. For lysine conjugation on CD3 antibody, zero to three bridge-DNA molecules were attached to each antibody, and for Glyclick conjugates on CD8a antibody, one or two bridge-DNA molecules were attached to each antibody. **c**, Agarose gel analysis of synthesized concatemer (bc42–19*_p.50, see Supplementary Table 1 for sequence) with different concentrations of hairpin templates used for the primer exchange reaction for concatemer synthesis. The product formed with 1 µM of the hairpin yielded a concatemer of the desired length of 650 nucleotides. **d**, Schematic showing isotope modification of imager DNA. Isotope-loaded polymer was conjugated to thiol-modified imager DNA. **e**, Agarose gel analysis of synthesized imager DNA (i.25-Yb172, see Supplementary Table 1 for sequence) before and after isotope labelling. Source data

In the SABER-IMC workflow, each antibody is tagged with a unique 42-mer bridge DNA. Each bridge DNA is then hybridized to the 5′ end of a single-stranded DNA concatemer (Extended Data Fig. 1a,b). The concatemer carries multiple repeats of a 9-mer barcode sequence at the 3′ end, which is then further hybridized to tens of imager strands, each conjugated to a metal isotope (Fig. 1a and Extended Data Fig. 1c–e). By using orthogonal bridge DNA sequences and 9-mer barcode sequences for different antibodies, signals of multiple targets can be simultaneously amplified. Additional rounds of concatemer hybridization, which create a branched DNA structure with additional imager binding sites, can further enhance the amplification^13^ (Fig. 1b). Here we use the terminology SABER×1, SABER×2 and SABER×3 to indicate one, two and three rounds of concatemer hybridization, respectively.

**Extended Data Fig. 2:**
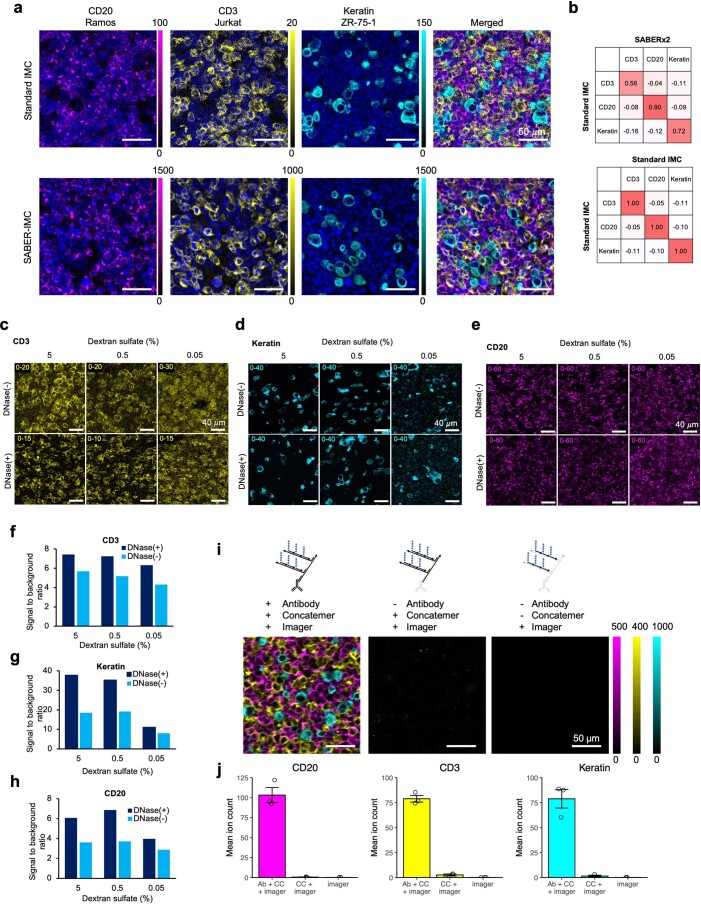
Validation and optimisation of SABER-IMC in mixed cell pellet. **a**, Representative standard IMC (top row) and SABER-IMC (bottom row) images of three ROIs obtained from an FFPE section of a cell pellet composed of Ramos, Jurkat, and ZR-75-1 cells stained for three markers. CD20 is shown in magenta, CD3 in yellow, and keratin in cyan. Intensities were scaled to show clear expression for each marker as indicated on the right of each image. Scale bars are 50 µm. **b**, Standard IMC and SABER-IMC were simultaneously performed on the same section of a mixed cell pellet. Top: Pixel-wise correlation of different markers between standard IMC and SABER×2. Bottom: Pixel-wise correlation comparing standard IMC against itself to show the expected marker correlation in each sample. **c-e**, Representative SABER-IMC images of **c)** CD3, **d)** keratin, and **e)** CD20 in FFPE section from a cell pellet composed of Ramos, Jurkat, and ZR-75-1 cells. Antibody solutions containing different concentrations of dextran sulfate with or without DNase treatment before antibody incubation were tested. Intensity scale is indicated on left top corner of each image. Scale bars are 40 µm. **f-h**, Signal-to-background ratios for **f**) CD3, **g**) keratin, and **h**) CD20. Signal intensity and background intensity were obtained from average intensity of pixels expressing higher or lower signal than defined threshold, respectively. **i**, Representative IMC image of three ROIs of SABER-IMC (left), SABER-IMC without antibody incubation (middle), and SABER-IMC with only imager incubation (right) images of FFPE sections of a cell pellet composed of Ramos, Jurkat, and ZR-75-1 cells stained for CD20 (magenta), CD3 (yellow), and keratin (cyan). Intensity scales are indicated on the right of the images. Scale bars are 50 µm. **j**, Signal intensity of CD20 (left), CD3 (middle), and keratin (right) for each condition described in panel **i**. Mean signal intensity was obtained using all pixels within each image. Error bars are s.e.m. (n = 3 independent experiments; each experiment is a mean value of three ROIs).

We first examined the specificity of the SABER-amplified signal using a mixed pellet of three different cell lines, Jurkat, Ramos and ZR-75-1 cells, which uniquely express CD3, CD20 and keratin, respectively. We performed SABER-IMC on formalin-fixed paraffin-embedded (FFPE) sections of the mixed cell pellet with antibodies to CD3, CD20 and keratin tagged with unique bridge DNAs. Reagents for the different targets (that is, antibodies, concatemers, imagers) were pooled before incubation. As a positive control, we performed standard IMC on a section from the same pellet stained with isotope-labeled primary antibodies^11^. We performed SABER amplification as previously reported^13^, with some modifications. We observed expression of the expected marker in each cell line (Extended Data Fig. 2a), as confirmed by pixel-wise correlation for simultaneous IMC and SABER-IMC staining of the same section (Extended Data Fig. 2b). A dextran sulfate concentration of 0.5–5% in the antibody solution and DNase pretreatment of the cell pellet reduced the nonspecific background, and concatemers and imagers did not introduce notable background (Extended Data Fig. 2c–j).

**Extended Data Fig. 3:**
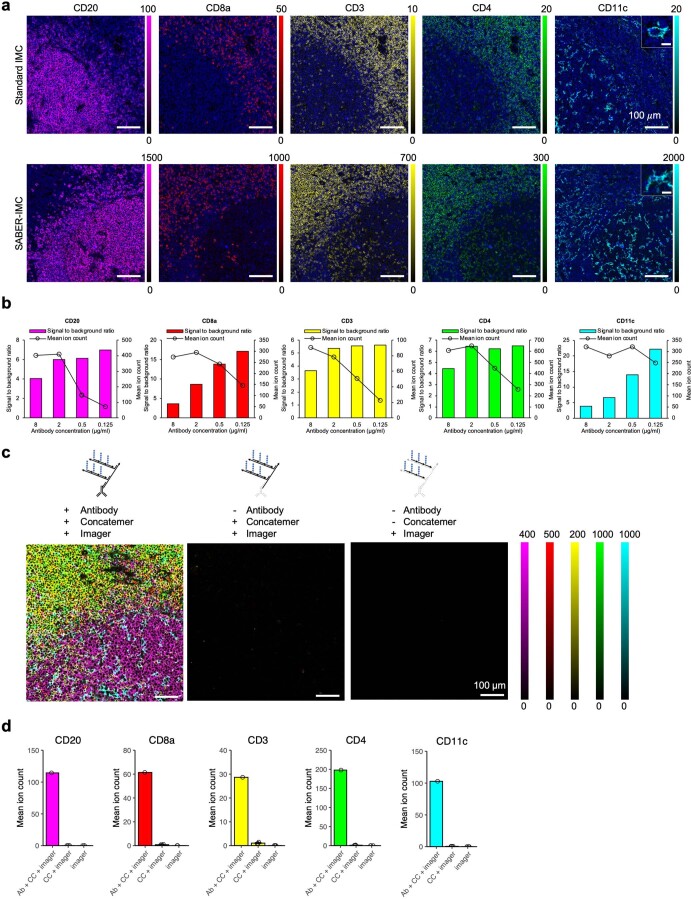
Validation and optimisation of SABER-IMC in tonsil. **a**, Images of single ROIs obtained from FFPE sections of human tonsil stained for CD20 (magenta), CD8 (red), CD3 (yellow), CD4 (green), and CD11c (cyan) imaged with standard IMC (top row) and SABER-IMC (bottom row). Top-right corner of CD11c image is a magnified view of a CD11c^+^ cell from the region indicated by a white box. Nuclear marker staining is displayed in blue in all panels. Intensities were scaled to show clear expression for each marker as indicated on the right of each image. Scale bars are 100 µm with the exception of the image in the magnified view which is 10 µm. **b**, Signal-to-background ratio and signal intensity in 5-plex SABER-IMC of tonsil for varying concentrations of antibodies targeting CD20, CD8a, CD3, CD4, and CD11c. Signal intensity and background intensity were obtained from average intensity of pixels expressing higher or lower signal than defined threshold, respectively. **c**, Images of single ROIs obtained from FFPE sections of tonsil stained for CD20 (magenta), CD8 (red), CD3 (yellow), CD4 (green), and CD11 (cyan) imaged with SABER-IMC (left), SABER-IMC without antibody incubation (middle), and SABER-IMC with only imager incubation (right). Intensity scales are indicated on the right of the images. Scale bars are 100 µm. **d**, Signal intensity for CD20, CD8a, CD3, CD4, and CD11c for each condition described in panel **c**. Mean signal intensity was obtained using all pixels within each image. Error bars for ‘CC+imager’ and ‘imager’ are s.e.m. (n = 3 independent experiments; each experiment is a mean value of single ROI).

We further tested the specificity of 5-plex SABER-IMC on FFPE human tonsil tissue, which has well-characterized protein expression patterns^14^ (Extended Data Fig. 3a). We selected antibody concentrations to maximize signal intensity while maintaining a signal-to-background ratio comparable to that of standard IMC (Extended Data Fig. 3b). Concatemers and imagers did not introduce notable background (Extended Data Fig. 3c,d), and expression of markers CD20, CD8a, CD3, CD4 and CD11c in tonsil were comparable in SABER-IMC and standard IMC (Extended Data Fig. 3a).

**Extended Data Fig. 4:**
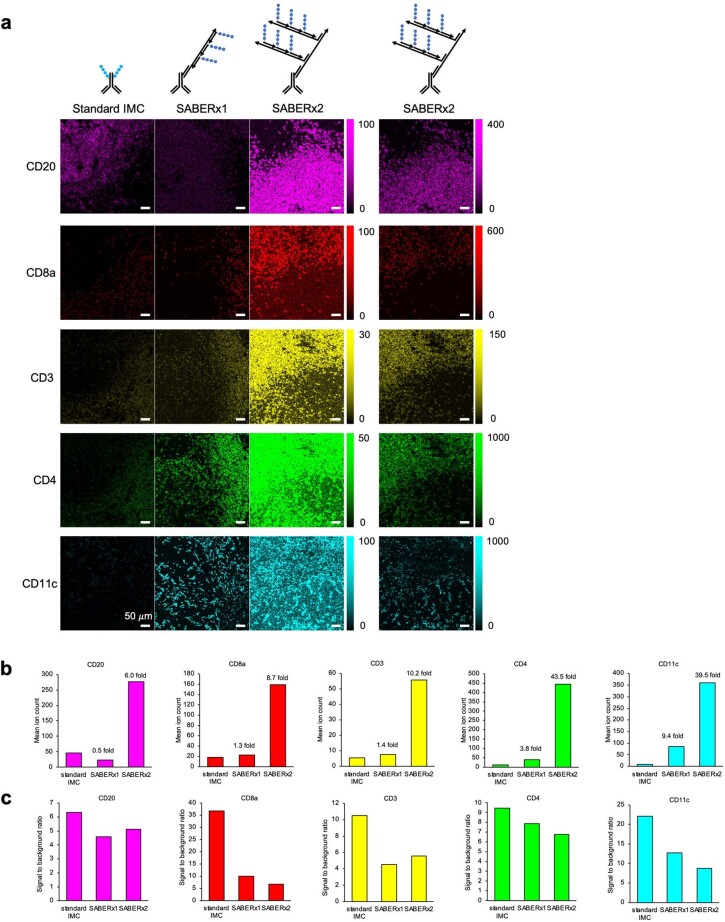
Quantification of SABER-IMC signal amplification in tonsil. **a**, Representative SABER-IMC images of two ROIs obtained from tonsil stained for CD20 (magenta), CD8a (red), CD3 (yellow), CD4 (green), and CD11c (cyan) with the indicated amplification and with standard IMC. Intensity scales are indicated on the right of images. Scale bars are 50 µm. **b**, Bar plots showing the mean signal intensities of CD20, CD8a, CD3, CD4, and CD11c. Fold amplification compared to standard IMC is indicated on top of each bar. Mean signal intensity was obtained using pixels expressing higher signal than defined threshold. **c**, Signal-to-background ratios for CD20, CD8a, CD3, CD4, and CD11c. Signal intensity and background intensity were obtained from average intensity of pixels expressing higher or lower signal than defined threshold, respectively.

To quantify signal amplification, we measured the signal intensity in mixed cell pellet images from SABER×1, SABER×2 and SABER×3 and compared with standard IMC and IMC amplified via isotope-labeled secondary antibodies. For CD3, SABER×1 amplified the signal 2.0-fold, SABER×2 16.7-fold and SABER×3 68.0-fold compared to standard IMC, with only very minor decrease in signal-to-background ratio (Fig. 1c–e). For CD20 and keratin, the signals were also increasingly amplified by the SABER×1, ×2 and ×3 relative to standard IMC, with 16.7-fold and 25.4-fold amplification for SABER×3 for the two proteins, respectively (Fig. 1c,d). Compared to IMC amplified via isotope-labeled secondary antibodies, SABER improved amplification by between 2- and 17-fold (Fig. 1c,d). We observed similar results in FFPE sections of tonsil (Extended Data Fig. 4).

**Fig. 2:**
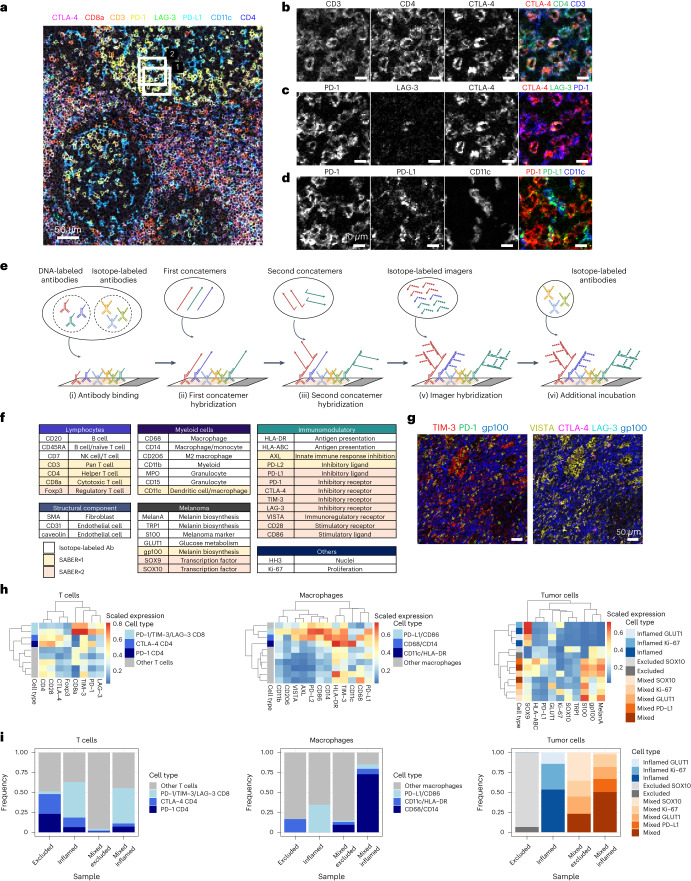
Detection of low-abundance molecules with SABER-IMC. **a**, SABER-IMC image of FFPE tonsil section stained for four low-abundance (CTLA-4, PD-1, LAG-3, PD-L1) and four high-abundance (CD3, CD4, CD8a, CD11c) markers. Scale bar, 50 µm. **b**, Magnified views of white box 1 in **a** showing the indicated markers (CD3, CD4, CTLA-4). Scale bars, 10 µm. **c**, Magnified views of white box 1 in **a** showing the indicated markers (PD-1, LAG-3, CTLA-4). Scale bars, 10 µm. **d**, Magnified view of white box 2 in **a** showing the indicated markers (PD-1, PD-L1, CD11c). Scale bars, 10 µm. **e**, Schematic of imaging of high-abundance targets with standard IMC and low-abundance targets with SABER-IMC. DNA-labeled antibodies for SABER amplification are incubated together with isotope-labeled antibodies. Concatemer and imager hybridization are performed as for the SABER-IMC protocol. **f**, Antibody panel used for 38-plex SABER-IMC of human melanoma. Target molecules and cell type or functional annotation information are listed. **g**, Representative SABER-IMC image of the indicated immunomodulatory molecules in inflamed melanoma tissue. gp100 is visualized in both images to indicate melanoma region. Scale bars, 50 µm. **h**, PhenoGraph clustering for T cells (left), macrophages (middle) and tumor cells (right) on the basis of their single-cell molecular expression. Clusters were manually annotated on the basis of expression pattern. **i**, Frequencies of cell phenotypes within the total population of T cells (left), macrophages (middle) and tumor cells (right) in excluded, inflamed and mixed melanoma samples. The mixed sample was further separated into ‘mixed excluded’ and ‘mixed inflamed’ regions, depending on the inflammation status of specific regions within the same sample.

**Extended Data Fig. 5:**
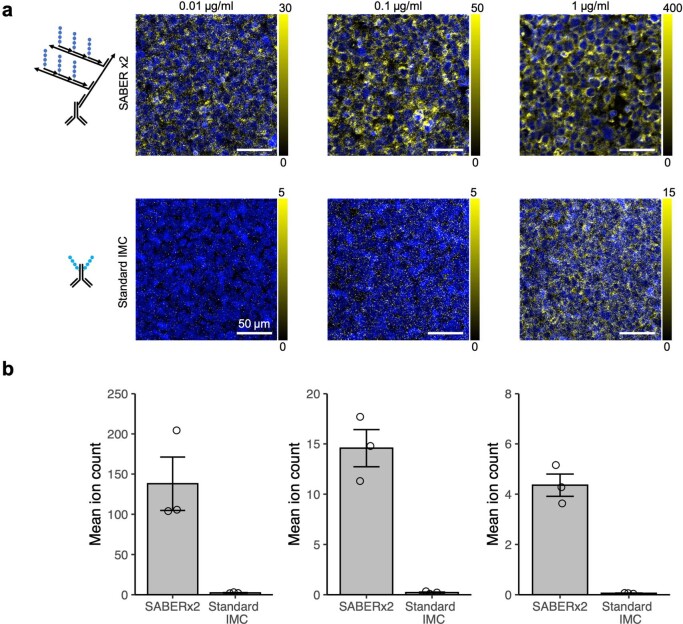
Detection of low concentration of antibodies by SABER-IMC. **a**, Representative SABER×2 (top row) and standard IMC (bottom row) images of three ROIs obtained from Jurkat cell pellet stained for CD3 with different antibody concentrations (0.01, 0.1, 1 µg ml^−1^). Intensity scales are indicated on the right of each image. Scale bars are 50 µm. **b**, Bar plots showing the mean signal intensity for each antibody concentration. Mean signal intensity was obtained using all pixels within each image. Error bars are s.e.m. (n = 3 independent experiments; each experiment is a mean value of three ROIs).

**Extended Data Fig. 6:**
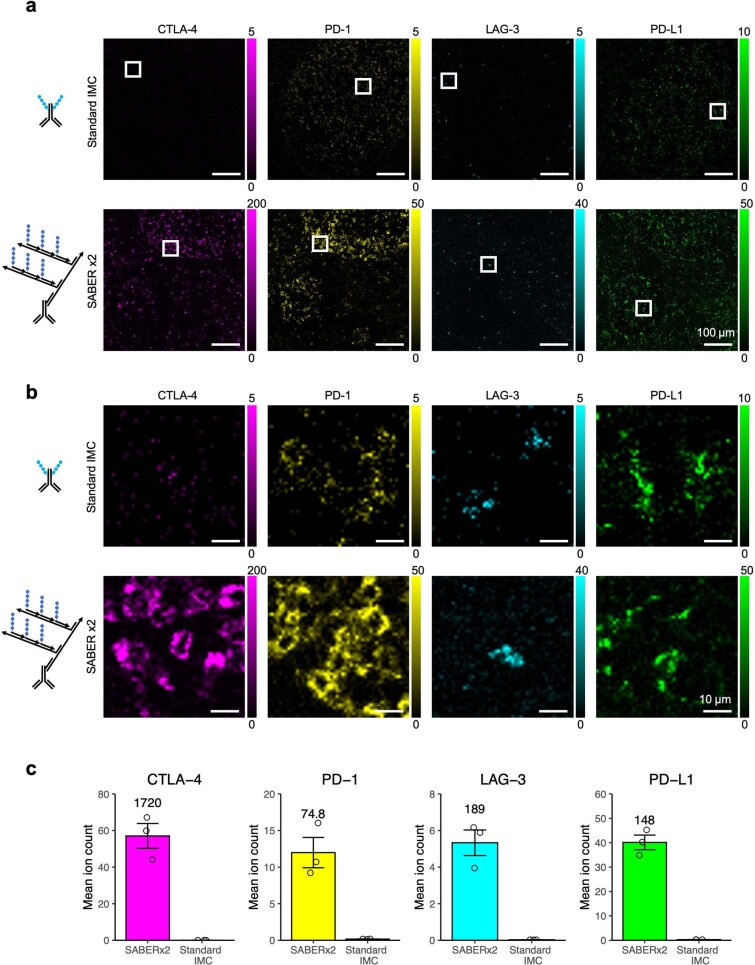
Detection of low-abundance markers in tonsil by SABER-IMC. **a**, Representative standard IMC (top row) and SABER×2 (bottom row) images of four ROIs obtained from FFPE sections of tonsil stained for low-abundance markers CTLA-4, PD-1, LAG-3, and PD-L1. Intensity scales are shown on the right of the image. Note that the scale is different for each comparison and that the display intensity is higher for SABER×2 in all cases. White box within the image indicates the position of magnified view in panel **b**. Scale bars are 50 µm. **b**, Magnified view of images in panel **a**. Scale bars are 10 µm. **c**, Bar plots showing the mean signal intensity for each target in standard IMC or SABER×2. Mean signal intensity was obtained using all pixels within each image. Fold amplification compared to standard IMC is indicated on top of each bar. Error bars are s.e.m. (n = 3 independent experiments; each experiment is a mean value of single ROI).

To model SABER-IMC detection of a low-abundance protein, we prepared CD3 antibody dilution series and compared the SABER-IMC and standard IMC signal on Jurkat cell pellet sections. At the lower antibody concentrations (0.01–0.1 µg ml^−1^), standard IMC detected no or very low CD3 signal, whereas the signal was detectable in SABER×2 (Extended Data Fig. 5). Next, we tested 8-plex SABER-IMC on human tonsil-targeting immune cell markers (CD3, CD4, CD8a and CD11c) and low-abundance immunomodulatory molecules (CTLA-4, PD-1, LAG-3 and PD-L1). These low-abundance proteins are impossible or difficult to detect by standard IMC. All markers were detected with SABER-IMC with the expected expression patterns^15–18^ (Fig. 2a and Extended Data Fig. 6). CTLA-4 expression was mainly found on CD3^+^/CD4^+^ T cells and was coexpressed with PD-1 and LAG-3 on only a subset of T cells (Fig. 2b,c). PD-L1 was mainly expressed on CD11c^+^ macrophages or dendritic cells, which were often found in the proximity of PD-1^+^ T cells (Fig. 2d), suggesting the expected PD-1/PD-L1 interaction^19,20^.

**Extended Data Fig. 7:**
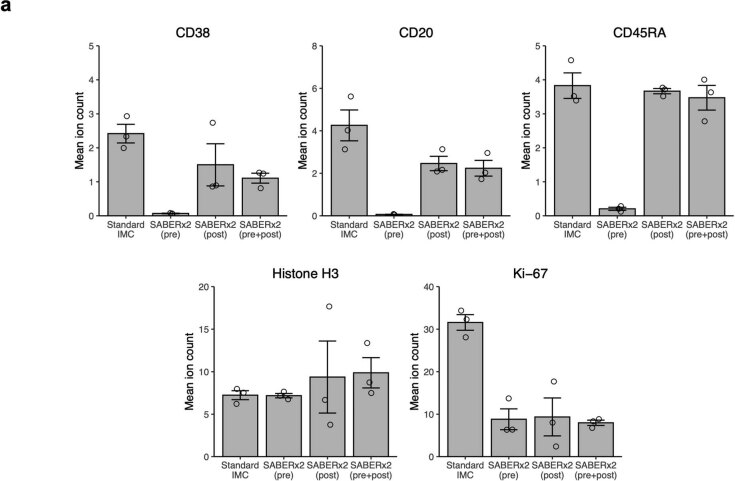
Intensity of isotope-labeled antibodies after SABER-IMC. **a**, Signal intensity of isotope-labeled antibodies for the indicated markers in sections of a Ramos cell pellet stained with standard IMC, SABER×2(Pre) (that is, isotope-labeled antibodies were incubated with tissue following the SABER×2 protocol), SABER×2(Post) (that is, first performed SABER×2 protocol without isotope-labeled antibodies, then incubated with isotope-labeled antibodies), SABER×2(Pre+Post) (that is, isotope-labeled antibodies were incubated with tissue following the SABER×2 protocol, and then tissues were incubated with isotope-labeled antibodies again). Mean signal intensity was obtained using all pixels within each image. Error bars are s.e.m. (n = 3 independent experiments; each experiment is a mean value of three ROIs).

Next, to detect targets with a large range of abundance, we imaged high-abundance targets with standard IMC and low-abundance targets with SABER-IMC (Fig. 2e). Antibodies for both strategies were mixed and simultaneously imaged. On Ramos cell pellet sections, the standard IMC signal was decreased by 25–99%, depending on the marker, by the SABER amplification steps. This was minimized to 0–70% by incubating tissues with isotope-labeled antibodies after the SABER amplification step (Extended Data Fig. 7).

**Extended Data Fig. 8:**
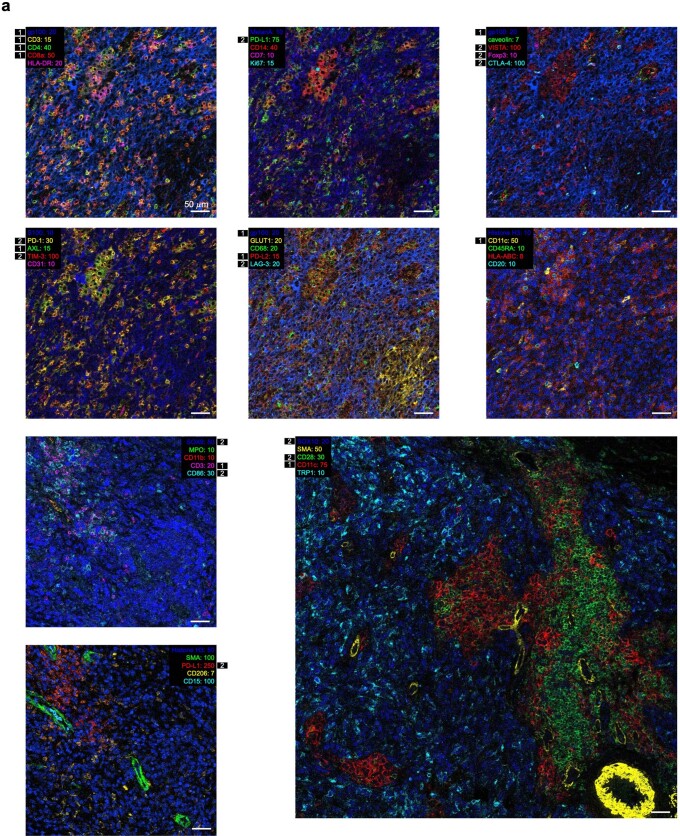
Example images for the indicated markers from a 38-plex imaging of human melanoma tissues. Degree of amplification for each marker is indicated in white next to its marker name: 1, SABER×1; 2, SABER×2; no label, standard IMC). Coloured number X indicated for each marker means its intensity scale of 0 - X. Scale bars are 50 µm.

**Extended Data Fig. 9:**
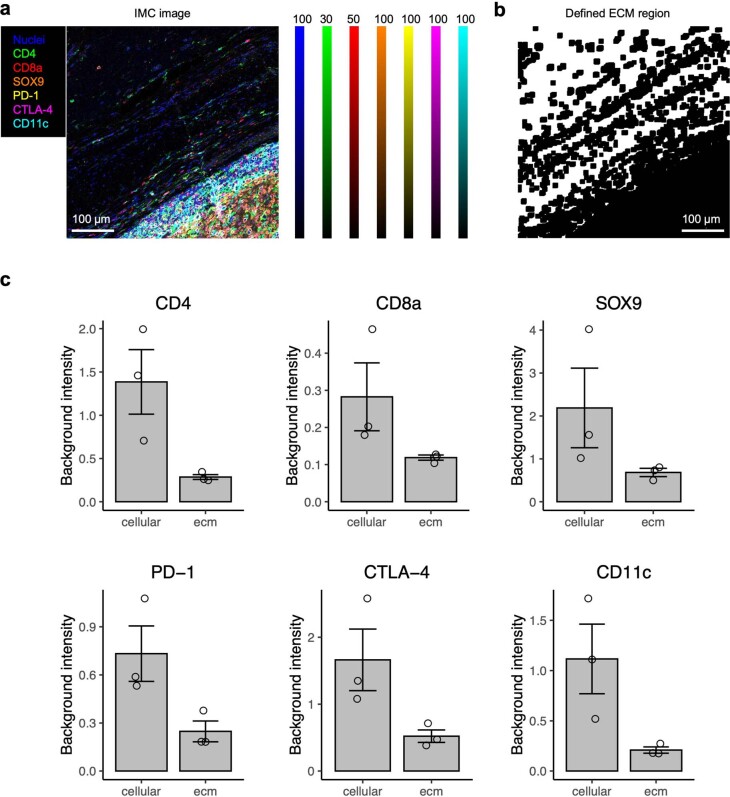
Background signal from extracellular matrix of human melanoma tissues. **a**, Representative SABER-IMC image of three ROIs used for the quantification of background signal from extracellular matrix (ECM) of human melanoma tissues. Nuclear marker staining is displayed in blue. Intensity scales are shown on the right of the image. Scale bars are 100 µm. **b**, Defined ECM region of the image in panel **a**. ECM region was defined as outside of cellular region. Cellular region was defined as cell mask expanded for 5 µm from the border to ensure no cellular signal would be observed in defined ECM region. **c**, Background intensities of ECM region and cellular region for CD4, CD8a, SOX9, PD-1, CTLA-4 and CD11c. Background intensity for ECM region was obtained from average intensity of pixels in ECM region. Background intensity for cellular region was obtained from average intensity of pixels in cellular region expressing lower signal than defined threshold. Error bars are s.e.m. (n = 3 ROIs from single experiment).

Using this approach, we applied SABER-IMC to three melanoma samples differing in their inflammation state to spatially analyze the TME. In the inflamed tumor, immune cells have infiltrated into the tumor mass. In the excluded tumor, immune cells remain outside the tumor mass. The mixed tumor contains both inflamed and excluded regions. We designed an antibody panel with 7 antibodies to be detected by SABER×1, 11 by SABER×2 and 20 by standard IMC (Fig. 2f). This panel included antibodies targeting many low-abundance immunomodulatory molecules that are difficult or impossible to detect by standard IMC. SABER-IMC detected all markers, including the low-abundance immunomodulatory molecules (Fig. 2g and Extended Data Fig. 8), with low background in extracellular matrix regions (Extended Data Fig. 9).

**Extended Data Fig. 10:**
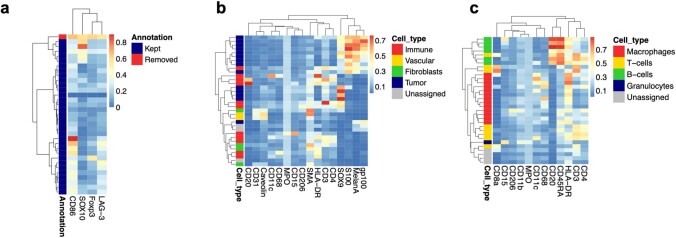
Highly multiplexed SABER-IMC reveals tumour-immune microenvironment characteristics of melanoma. **a**, PhenoGraph clustering of expression patterns of CD86, LAG-3, Foxp3, and SOX10. These markers are not expected to be co-expressed within any cell and were used to identify and remove nonspecific signal. **b**, PhenoGraph clustering of 38-plex SABER-IMC-derived expression patterns in all cells in the melanoma samples after the removal of nonspecific signal. Clusters were manually annotated as tumour cells, immune cells, vascular cells, fibroblasts, or unassigned cells based on expression profiles. **c**, PhenoGraph clustering of all immune cells in the melanoma samples. Clusters were manually annotated as macrophages, T cells, B cells, or granulocytes based on expression profiles.

To determine the phenotypes of individual cells within the TMEs, we segmented single cells^21^, removed rare cell populations that nonspecifically accumulated SABER-IMC signal especially from SABER×2 amplified targets (Extended Data Fig. 10a), and then clustered and annotated all cells as T cells, macrophages, tumor cells and other cells on the basis of known marker expression (Extended Data Fig. 10b,c). We identified two important phenotypes within the T cell population: CTLA-4^+^/Foxp3^+^ regulatory T cells, which have been previously identified in melanoma^22^, and PD-1^+^/LAG-3^+^/TIM-3^+^ CD8^+^ T cells (Fig. 2h). CTLA-4^+^ regulatory T cells were found in both the excluded and the inflamed samples (Fig. 2i). PD-1^+^/LAG-3^+^/TIM-3^+^ CD8^+^ T cells were mainly found in the inflamed sample and in inflamed regions of the mixed sample (Fig. 2i), as expected^23^. We also identified distinct clusters of macrophages and tumor cells (Fig. 2h,i). These experiments demonstrate that SABER-IMC enables the imaging of low-abundance immunomodulatory molecules within the context of a highly multiplexed experiment, allowing for single-cell phenotypic and spatial analysis of the TME.

SABER-IMC is the first highly multiplexed signal amplification method for IMC. Depending on the abundance of a target within the sample, no amplification or up to three rounds of amplification can be used, allowing tissue imaging across a wide abundance range, which would not have been possible with existing approaches. SABER-IMC amplified signal 8- to 16-fold with two rounds of amplification, and 16- to 68-fold with three rounds of amplification, compared to standard IMC; the extent of amplification was target dependent. This variation could result from the antibody conjugation method, concatemer length or oligonucleotide sequence. Taking mRNA abundance as a proxy for protein abundance^24^, although we recognize that this can be imperfect for many markers, we achieved the simultaneous detection of as low as 40 normalized transcripts per million (nTPM) up to 5,300 nTPM (for example, 41.9 nTPM for CTLA-4, 5,290.7 nTPM for SMA) (ref. ^24^). Finally, SABER-IMC facilitates IMC panel design, since less sensitive channels can be used for SABER-amplified markers.

SABER-IMC combines two technologies, immuno-SABER and IMC, and retains the advantages of both while compensating for their weaknesses. Immuno-SABER enables highly multiplex imaging with signal amplification, but it requires repetitive imager hybridization cycles, which are eliminated in SABER-IMC. Our simultaneous 38-plex SABER-IMC imaging would require 13 cycles with three colors per round using immuno-SABER, including sample registration. Additionally, SABER-IMC allows inclusion of isotope-labeled primary antibodies, and is thus more flexible than immuno-SABER at reduced cost and effort of reagent synthesis. Finally, although several DNA-based signal amplification methods have been reported for immunofluorescence imaging^25,26^, we chose SABER because it was the only high multiplexity (10-plex) method reported so far, and avoiding in situ amplification should improve reproducibility.

We used 29 out of 50 barcoded DNA sequences from a previous publication^13^ in our 18-plex SABER panel (7-plex SABER×1 and 11-plex SABER×2) for melanoma samples. Higher multiplexity may require additional design and validation of DNA barcodes. Potentially, longer barcode sequences than the current 9-mer barcodes could help to generate larger numbers of orthogonal DNA barcodes. When conjugating the bridge DNA to antibodies, we recommend using site-specific conjugation approaches (for example, targeting glycans at the antibody Fc region) over non-site-specific approaches (for example, targeting lysine residues on an antibody). Site-specific DNA conjugation minimizes the interference with antigen recognition of the conjugated antibody and eliminates the need for optimizing reaction conditions (for example, concentrations of cross-linking molecule and bridge DNA) for each antibody to yield 1–3 DNA conjugations per antibody, as suggested previously^13^.

In summary, SABER-IMC is a highly multiplexed amplification platform for high-resolution tissue imaging, which allows detection of low-abundance targets that are impossible or difficult to detect with standard IMC. SABER-IMC does not require sequential hybridization cycles, and we report much higher multiplexing than demonstrated for other signal-amplification technologies (10-plex for immuno-SABER, 8-plex for IHC-TSA^27^). As demonstrated in our analysis of melanoma samples, SABER-IMC enabled clear detection of low-abundance immunomodulatory molecules and should prove useful for numerous other classes of low-abundance proteins, such as transcription factors. SABER-IMC is a powerful technique for highly multiplexed tissue imaging as demonstrated here and in the accompanying manuscript (Strotton et al.^28^).

## Methods

### Antibody labeling with bridge-DNA tags

Antibody and bridge DNA were conjugated either via click reaction (azide-modified antibody and DBCO-modified DNA) or via thiol–maleimide addition reaction (maleimide-modified antibody and thiol-modified DNA) (Extended Data Fig. 1a). DNA sequences of all the bridge DNA identities (IDs) are listed in Supplementary Table 1. Details of the antibodies and the corresponding bridge DNA IDs are in Supplementary Table 2. Conjugation via thiol–maleimide addition reaction was used for anti-CD3 and for anti-keratin. All other antibodies were conjugated via click reaction. For the click reaction, antibodies were modified with azide using Glyclick azide activation kit (Genovis) following the protocol from the manufacturer. Briefly, glycans in the Fc region of antibodies were enzymatically truncated and remodified with *N*-azidoacetylgalactosamine. The product was purified using 0.5 ml, 50 kDa Amicon Ultra Filters (Millipore). Next, 10 molar equivalents of 5′-DBCO-modified DNA (Microsynth) was added to 1 mg ml^−1^ of azide-modified antibody in PBS solution and kept at room temperature overnight to complete the click reaction. Conjugated antibody was purified with 0.5 ml, 50 kDa Amicon Ultra Filters.

For thiol–maleimide addition reaction, lysine residues on antibodies were modified with maleimide using maleimide-PEG_2_-NHS cross-linker (Thermo Fisher Scientific)^13,29^. Antibodies were concentrated to around 2 mg ml^−1^ in PBS using 0.5 ml, 50 kDa Amicon Ultra Filters, then 13–65 molar equivalents of maleimide-PEG_2_-NHS cross-linker was added. Reactions were kept at 4 °C for 2 h. Maleimide-modified antibodies were purified over 0.5 ml, 7 kDA Zeba desalting columns (Thermo Fisher Scientific). In parallel, 100 μM of 5′-thiol-modified bridge DNA was activated with 20 mM TCEP in water for 30 min at 37 °C, then purified using a NAP5 column (GE Healthcare Life Sciences). Next, 10 molar equivalents of activated 5′-thiol-modified DNA was added into the solution of maleimide-modified antibody in PBS, and the reaction was kept at 4 °C overnight. Conjugated antibody was purified over 0.5 ml, 50 kDa Amicon Ultra Filters.

Obtained DNA–antibody conjugates were analyzed by gel electrophoresis. Antibodies were denatured in LDS buffer (Thermo Fisher Scientific) at 95 °C for 3 min without reducing reagents and run on 4–12% Bis–Tris Plus gels (Thermo Fisher Scientific) at 165 V for 60 min. The gels were stained with SimplyBlue safe stain (Thermo Fisher Scientific) for 1 h and washed with deionized water for 1 h to overnight before visualization. Example gel analysis results of DNA-conjugated antibodies are shown in Extended Data Fig. 1b.

### Antibody labeling with metal isotope

Isotope-labeled antibodies were used for standard IMC staining and for unamplified targets. For secondary antibody amplification, intact primary antibody and isotope-labeled secondary antibody were used. Isotope labeling of antibodies was carried out using the MaxPar X8 antibody labeling kit (Fluidigm) according to the manufacturer’s manual. First, chelation was completed by incubating MaxPar X8 polymer, which carries terminal maleimide functionality and multiple chelators for lanthanide ions, in 2.5 mM lanthanide chloride solution (Fluidigm) at 37 °C for 30 min. The product was purified into C buffer, provided with the MaxPar X8 antibody labeling kit, using 0.5 ml, 3 kDa Amicon Ultra Filters (Millipore). In parallel, antibody was partially reduced in 0.8 mM TCEP at 37 °C for 30 min and was purified into C buffer using 0.5 ml, 50 kDa Amicon Ultra Filters. Isotope-loaded polymer was added into partially reduced antibody and was incubated at 37 °C for 90 min. Conjugated product was purified over 0.5 ml, 50 kDa Amicon Ultra Filters. Details of the antibodies and the corresponding metal isotope modifications are in Supplementary Table 2.

### PER concatemer synthesis for SABER amplification

All concatemers were synthesized as reported previously with minor changes^13,29^. Before adding primers, the reaction mixture was prepared with final concentrations of 800 U ml^−1^ of BST LF polymerase (NEB), 600 μM each of dATP, dTTP and dCTP (NEB), 0.1 to 2 µM of hairpin (IDT), 100 nM of Clean.G hairpin (5′-CCCCGAAAGTGGCCTCGGGCCTTTTGGCCCGAGGCCACTTTCG-3′, IDT) and 10 mM MgSO_4_ (NEB) in PBS. The reaction mixture was preincubated at 37 °C for 15 min to remove potentially contaminating dGTP, which could inhibit the reaction. Subsequently, primers were added to a final concentration of 1 µM, and the reaction was kept at 37 °C or 25 °C for 2–24 h, followed by heat inactivation of polymerase at 80 °C for 20 min. In addition to optimizing hairpin concentration and reaction time, we decreased reaction temperature from 37 °C to 25 °C for some of the primers to allow extension of up to 650 nucleotides in length. DNA sequences of all the primers and concatemers are listed in Supplementary Table 1. Details of the reaction conditions for the concatemer synthesis are in Supplementary Table 3. Concatemers were either directly used without further purification or were purified and concentrated with a Minelute kit (Qiagen) into water. Concentration of concatemers is necessary when SABER-IMC is performed in more than 6-plex. We used the Minelute kit to concentrate 100 µl concatemer solution into 10 µl. Concatemer products and a 1 kilobase pair (kb) Plus DNA ladder (Thermo Fisher Scientific) were separated on 1% agarose gel electrophoresis in TAE buffer at 120 V for 40 min. The gel was stained with GelRed (Biotium) and visualized with a transilluminator (TFX-20, Sigma). An example gel analysis is shown in Extended Data Fig. 1c.

### Imager labeling

The MaxPar X8 antibody labeling kit (Fluidigm) was used to prepare metal isotope-modified polymer with a terminal maleimide functionality. Chelation was completed by incubating MaxPar X8 polymer in 2.5 mM lanthanide chloride solution (Fluidigm) at 37 °C for 30 min, and the product was purified into C buffer using 0.5 ml, 3 kDa Amicon Ultra Filters (Millipore). The 5′-thiol-modified imager DNA (Microsynth) was activated using 50 mM TCEP at 25 °C for 30 min, followed by the addition of sodium acetate to a final concentration of 0.3 M. After vortexing, an aliquot of 5 nmol of DNA (20 μl) was used for one tube of MaxPar X8 polymer. To the tube containing DNA, 2.5 volume equivalents of ethanol (−20 °C) was added. The sample was vortexed and incubated at −20 °C for 30 min. The reaction was centrifuged at 30,000*g* for 30 min at 4 °C. The pellet was washed with 500 µl 75% ethanol and centrifuged at 30,000*g* for 3 min at 4 °C. The precipitated product was obtained by removing the supernatant and resuspending into 50 µl C buffer. The product concentration was determined using a Nanodrop spectrophotometer. An aliquot of 1.5–2 nmol of activated 5′-thiol-modified imager DNA was added to purified isotope-labeled MaxPar X8 polymer in 200 µl C buffer and incubated at 25 °C for 2.5 h. The sample was purified three times into 40 µl water using 0.5 ml Microcon 30 filters (Millipore). TCEP was optionally added to a final concentration of 5 mM at 30 min before the purification step. DNA sequences of all the imagers are listed in Supplementary Table 1. The obtained isotope-labeled imager was analyzed on a 4% agarose gel run at 120 V for 40 min. Gels were stained with GelRed and visualized using a transilluminator. Examples are shown in Extended Data Fig. 1d,e.

### Preparation of FFPE sections of mixed cell pellet, human tonsil and melanoma tissue

Jurkat cells and Ramos cells were cultured and expanded in RPMI 1640 (Sigma) with 5% or 10% fetal bovine serum until the cell number reached around 160 million. ZR-75-1 cells were cultured and expanded in DMEM (Gibco) with 1 mM sodium pyruvate (Gibco) supplement until the cell number reached around 10 million. We then mixed 33 million Jurkat cells, 106 million Ramos cells and 7 million ZR-75-1 cells and washed the cells with PBS several times. Cells were then centrifuged and resuspended in human plasma (Sigma). Next, bovine thrombin was added, and the cell suspension was incubated at room temperature for 10 min until cells clotted. The cell pellet was transferred into a biopsy capsule, and cells were fixed with 10% formalin (Thermo Fisher Scientific) overnight. The pellet was washed with twice with PBS (15 min each wash), once with 50% ethanol (30 min) and once with 70% ethanol (overnight). Finally, the cell pellet was embedded in paraffin. FFPE sections of 6 µm were prepared using a rotary microtome. Sections were collected onto glass slides (J1800AMNZ, Thermo Fisher Scientific) and baked overnight at 37 °C. The FFPE sections were kept at 4 °C for short-term storage or at −20 °C for long-term storage. Human tonsil FFPE sections were prepared at University Hospital of Zurich. Human melanoma tissue FFPE section were prepared at the Department of Dermatology, University Hospital of Zurich. Samples received from University Hospital Zurich were approved by the Ethikkommission Kanton Zürich (KEK-ZH-Nr 2014-0425).

### General SABER-IMC protocol for FFPE sections

The SABER protocol was adopted and slightly modified from the literature^13,29^. FFPE sections were left at room temperature for 15 min after removal from storage. Deparaffinization was carried out using an AS-2 (Pathisto). The slides were placed into HIER buffer (10 mM Trizma (Sigma), 1 mM EDTA (Thermo Fisher Scientific), pH 9.2) and heated at 95 °C for 30 min in a decloaking chamber (BioCare Medical) for epitope retrieval. Slides were cooled at room temperature for 20 min, washed in PBS for 15 min and regions of interest were outlined with a hydrophobic pen (Vector Laboratories). Before staining, samples were incubated with 20 U ml^−1^ DNase I (NEB) in 1× DNase buffer (NEB) at 37 °C for 2 h, followed by washing with deionized water for a few seconds and with PBS (three times for 10 min each) at room temperature. DNase treatment helped to get rid of nonspecific nuclei signal observed in SABER-IMC. Samples were then blocked with PBS containing 3% BSA (Sigma) and 0.1% Triton X-100 (Sigma) for 1 h at room temperature in a humidified chamber.

Antibody solution was prepared in the blocking buffer (PBS containing 3% BSA (Sigma) and 0.1% Triton X-100 (Sigma)) containing 0.2 μg ml^−1^ sheared salmon sperm DNA (Thermo Fisher Scientific) and 0.5–5% dextran sulfate (Sigma) and was incubated at 4 °C overnight in a humidified chamber. Antibodies were titrated to give optimal signal-to-background ratio. Typically, antibodies were diluted to 0.1–2 μg ml^−1^ final concentrations. Excess antibodies were removed by washing three times for 10 min each in PBS, and remaining antibodies were cross-linked by addition of 5 mM α,ω-Bis-NHS-PEG (2,000 molecular weight, Sigma) in PBS at 4 °C for 3 h. Reactions were quenched in TBS at room temperature for 20 min.

Concatemer solution was prepared in 2× SSC buffer (Sigma) with 25% formamide (Sigma), 10% dextran sulfate, 0.1% (v/v) Tween-20 (Sigma) and 0.2 mg ml^−1^ sheared salmon sperm DNA. Concatemers were typically diluted to 80–100 nM, and concatemers for different targets were mixed together and used for simultaneous staining. Therefore, at multiplexity over 6-plex, it is necessary to concentrate concatemer solutions from the 1 μM PER reaction to 10 μM using the Minelute kit. At 10 μM stock concatemer solution, more than 40 different concatemers can be included in the total volume at 100 nM final concentration for each concatemer. The slides were incubated with concatemer solution at 37 °C for 1 h in a humidified chamber, then washed with 50% formamide in PBS for 5 min at room temperature and three times for 10 min each with TBS containing 0.1% Triton X-100 at 37 °C. For SABER×2 or ×3 amplification, concatemer solution was prepared in the same buffer, and slides were incubated in a humidified chamber at 37 °C for 2 h. Second- and third-round concatemers were typically diluted to 100 nM, and concatemers for different targets were mixed together and incubated simultaneously. Excess concatemers were removed by washing with 50% formamide in PBS for 5 min at room temperature and then three times for 10 min each with TBS containing 0.1% Triton X-100 at 37 °C.

Imager solution was prepared in PBS containing 0.1% Triton X-100, and slides were incubated in the solution at room temperature for 1 h in a humidified chamber. Imagers were typically diluted to 1 μM final concentration. After incubation, slides were washed once with PBS containing 0.1% Triton X-100 for 5 min at 37 °C and twice for 5 min each with TBS at room temperature. For nuclear staining, slides were incubated with 1:1,000 dilution of 500 µM MaxPar intercalator-Ir (Fluidigm) in PBS for 5–10 min, followed by a 15-min wash in TBS at room temperature. Slides were then dipped into deionized water for a few seconds, dried immediately using pressured air flow and stored at room temperature until measurements. Since iridium intercalator also interacts with SABER DNA constructs, cytosolic iridium signal was observed from SABER×2 and ×3 amplification for abundant targets, such as cytokeratin, when SABER-amplified signal counts were above 1,000. Typically, the iridium background signal for SABER×1 and SABER×2 is low and can be ignored. It is recommended, however, that an antibody-based nuclear stain, such as anti-histone H3, be used in experiments when SABER-amplified signal counts are above 1,000 and when the cytosolic background signal is an issue, for example, in nuclear segmentation.

IMC images were acquired using a Hyperion Imaging System (Fluidigm). The laser ablation frequency was at 200 Hz, and preprocessing of the raw data into mcd files was completed using commercially available acquisition software (Fluidigm). Automated tuning of the argon flow and helium flow was performed on a daily basis using a tuning slide coated with isotope-containing polymer (Fludigm).

### DNA barcode sequences and corresponding antibodies and metal isotopes

For all the experiments, details of bridge DNAs, concatemers, imagers and corresponding metal isotopes are given in Supplementary Table 4.

### SABER-IMC optimization on mixed cell pellet and human tonsil tissue FFPE sections

To minimize nonspecific nuclei signal, dextran sulfate and DNase were used. Deparaffinization, epitope retrieval, antibody staining, concatemer hybridization, imager hybridization and IMC acquisition were performed according to the general SABER-IMC protocol with minor changes. For the conditions without DNase treatment, samples were directly blocked with PBS containing 3% BSA and 0.1% Triton X-100 after epitope removal and washing. Dextran sulfate concentrations tested were 0.05%, 0.5% and 5%. For each condition, we performed SABER×1 with antibodies to CD20, CD3 and keratin. Details of bridge DNAs, concatemers and imagers are in Supplementary Table 4.

To optimize antibody concentrations, experiments were performed on human tonsil sections. Deparaffinization, epitope retrieval, antibody staining, concatemer hybridization, imager hybridization and IMC acquisition were performed according to the general SABER-IMC protocol except that antibody concentrations were titrated from 0.125 µg ml^−1^ to 8 µg ml^−1^. For each antibody concentration, we performed SABER×2 with antibodies to CD20, CD3, CD11c, CD8a and CD4. Details of bridge DNAs, concatemers and imagers are in Supplementary Table 4.

### Standard IMC protocol for mixed cell pellet and human tonsil tissue FFPE sections

FFPE sections were left at room temperature for 15 min after removal from storage. Deparaffinization was carried out using an AS-2 (Pathisto). The slides were placed into HIER buffer and heated at 95 °C for 30 min in a decloaking chamber (BioCare Medical) for epitope retrieval. Slides were cooled at room temperature for 20 min, washed in PBS for 15 min and ROIs were outlined with a hydrophobic pen (Vector Laboratories). Samples were then incubated with blocking buffer for 1 h at room temperature in a humidified chamber. Isotope-labeled antibody diluent was prepared in blocking buffer and incubated overnight at 4 °C. Details of antibodies and their isotope labels are in Supplementary Table 2. Samples were washed in TBS for 15 min and incubated with 1:1,000 dilution of 500 µM MaxPar intercalator-Ir (Fluidigm) in PBS for 5–10 min, followed by a 15-min wash in TBS at room temperature. Slides were then dipped into deionized water for a few seconds, dried immediately using pressured air flow and stored at room temperature until measurements.

### Secondary antibody amplification for mixed cell pellet FFPE sections

Deparaffinization, epitope retrieval and blocking were carried out as described in the standard IMC protocol. Intact primary antibody was incubated overnight at 4 °C, followed by two 10-min washes in TBS. Isotope-labeled secondary antibody was added, and samples were incubated at room temperature for 1 h then washed with TBS for 15 min at room temperature. Details of antibodies and their isotope labels are in Supplementary Table 2. For nuclear staining, slides were incubated with 1:100 dilution of 500 µM MaxPar intercalator-Ir (Fluidigm) in PBS for 5–10 min, followed by a 15-min wash in TBS at room temperature. Slides were then dipped into deionized water for a few seconds, dried immediately using pressured air flow and stored at room temperature until measurements.

### Quantification of signal intensity and signal-to-background ratio

Signal intensity was calculated as follows. First, the threshold intensity value was calculated for every image by robust background function in CellProfiler. Next, for each image, pixels with higher intensity than the threshold value were considered as signal regions, and the rest were considered as background. We confirmed that the number of pixels detected above the threshold did not change substantially between different conditions and visually inspected all thresholded images to ensure a lack of artifacts (for example, detecting background as a signal or vice versa). Finally, signal intensity was calculated by subtracting the average intensity of the background region from the average intensity of the signal region. The signal-to-noise ratio was obtained by dividing the average intensity of the signal region by average intensity of the background region. Note that we reported a slightly lower amplification fold than reported for immuno-SABER (5- to 20-fold and ~50-fold with one and two rounds of amplification, respectively^13,29^). This is probably because we compared the signal to that obtained in standard IMC using antibodies with up to four isotope labels each. In contrast, the amplification fold reported for immuno-SABER used the signal from a single imager binding site as the comparator. In cases where we did not detect a signal for standard IMC, it is not feasible to define a signal region. In these cases, we simply took the average signal intensity of all pixels within each image. Note that this quantification is susceptible to background signal and potential variation in the density of positive cell per image. For figures where we used this approach, we mention in the legend that the signal intensity was obtained from all pixels.

### Combining standard IMC antibodies to SABER-IMC

To assess whether SABER amplification affects the signal of simultaneously incubated isotope-labeled antibodies, we compared the intensity of isotope-labeled antibodies on the Ramos cell pellet using: (1) standard IMC; (2) SABER×2(Pre) (that is, isotope-labeled antibodies were incubated with tissue following the SABER×2 protocol); (3) SABER×2(Post) (that is, the SABER×2 protocol was performed without isotope-labeled antibodies, then tissue was incubated isotope-labeled antibodies following standard IMC protocol); and (4) SABER×2(Pre+Post) (that is, tissue was incubated with isotope-labeled antibodies following the SABER×2 protocol until the imager washing step, and then tissue was incubated with isotope-labeled antibodies again).

### SABER-IMC imaging of human melanoma FFPE sections for tumor immune microenvironment analysis

FFPE slides of tumors from three different patients with human melanoma were processed according to the general SABER-IMC protocol, with minor modification. During incubation with antibody–DNA conjugates, we also included isotope-labeled antibodies for highly abundant targets that do not require SABER amplification. After the SABER amplification protocol, additional incubation of isotope-labeled antibodies was performed. In total, 20 targets were imaged by isotope-labeled antibody, 7 targets were imaged by SABER×1 and 11 targets were imaged by SABER×2. Details of antibodies and their corresponding isotope labels or bridge DNAs, concatemers and imagers are in Supplementary Table 4.

### Data processing and single-cell signal quantification of 38-plex SABER-IMC images from human melanoma samples

Ion count image data from IMC software were transformed into tiff format and analyzed with an image-processing pipeline (https://github.com/BodenmillerGroup/ImcSegmentationPipeline). Briefly, Ilastik was used to train the pixel classifier by manually annotating pixels of random crop tiff images as nuclear, cytoplasmic or background on the basis of a combination of cytoplasmic or nuclear markers. The trained classifier was used to annotate all other pixels with probability values, providing the probability maps. The single-cell mask was generated on the basis of the probability maps using CellProfiler. Here, the nuclei were detected and cytoplasm identified by expanding the nuclei to the borders with the propagation method. Mean signal intensity of the individual cell area defined by this single-cell mask was used as the single-cell protein expression value. For subsequent PhenoGraph clustering, the single-cell protein expression data were arcsinh transformed and normalized to the 99.5th percentile.

### Removal of nonspecific spots

Rare cell populations in melanoma tissue nonspecifically accumulated SABER-IMC signal, especially for SABER×2 amplified targets. To remove these cells from analysis, all cells were classified into small clusters using PhenoGraph on the basis of four markers (CD86, LAG-3, Foxp3 and SOX10), which are not expected to be coexpressed within a cell. We removed the cells from the cluster that showed extremely high expression of all the four markers, as these regions nonspecifically accumulated SABER-IMC signal.

### Cell clustering

PhenoGraph was used for unsupervised clustering, and these clusters were aggregated and classified into cell types on the basis of median marker expression. The first PhenoGraph clustering for all the cells resulted in 21 clusters, classified into three general cell types (tumor cells, immune cells, vascular cells). Here we used 12 cell-type markers (SOX9, gp100, MelanA, CD31, SMA, caveolin, CD20, CD3, CD4, CD15, HLA-DR and CD11c) for the clustering. A second clustering on the cells classified as immune cells using 12 immune cell markers (CD3, CD8a, CD4, CD20, CD45RA, CD11b, CD15, MPO, CD14, CD68, HLA-DR and CD11c) resulted in 18 clusters that included T cells, macrophages, B cells and granulocytes. The T cell clusters, macrophage clusters and tumor cell clusters were characterized in detail using 8 markers for T cells (CD4, CD8a, CD28, Foxp3, PD-1, CTLA-4, TIM-3, LAG-3), 12 markers for macrophages (CD11b, CD68, CD206, CD14, CD11c, HLA-DR, CD86, PD-L1, PD-L2, AXL, VISTA, TIM-3) and 10 markers for tumor cells (SOX9, SOX10, gp100, MelanA, S100, HLA-ABC, PD-L1, TRP1, GLUT1, Ki-67).

### Statistics and reproducibility

Experiments for Fig. 1d,e and Extended Data Figs. 2j, 3d, 5b, 6c and 7 were performed in three experimental replicates. Other experiments were performed once.

### Reporting summary

Further information on research design is available in the Nature Portfolio Reporting Summary linked to this article.

## Online content

Any methods, additional references, Nature Portfolio reporting summaries, source data, extended data, supplementary information, acknowledgements, peer review information; details of author contributions and competing interests; and statements of data and code availability are available at 10.1038/s41592-023-01976-y.

## Supplementary information

Reporting Summary

Supplementary Tables Supplementary Table 1: DNA sequence corresponding to the ID of the DNA barcode. Supplementary Table 2: Antibody information. Supplementary Table 3: Reaction conditions for primer exchange reaction to synthesize concatemers. Supplementary Table 4: Antibody concentration, DNA barcode IDs and isotope mass tags used in each experiment.

## Data Availability

Tiff files for IMC data and single-cell data used in this manuscript are available on Zenodo (10.5281/zenodo.7942581). Source data are provided with this paper.

## Source data

Source Data Extended Data Fig. 1 Unprocessed gel images.

## Code availability

All code used for preprocessing and analyzing IMC data is available at https://github.com/BodenmillerGroup/SABER-IMC_publication.
