# Supplementary material for: DNA-barcoded signal amplification for imaging mass cytometry enables sensitive and highly multiplexed tissue imaging
Source: Nat Methods. 2023 Aug 31;20(9):1304–9. doi: 10.1038/s41592-023-01976-y (PMC10482679; doi:10.1038/s41592-023-01976-y)
Supplement: Supplementary file 1 — Reporting Summary [file 41592_2023_1976_MOESM1_ESM.pdf]

nature research

Corresponding author(s): Bernd Bodenmiller

Last updated by author(s): May 16, 2023

Reporting Summary

Nature Research wishes to improve the reproducibility of the work that we publish. This form provides structure for consistency and transparency in reporting. For further information on Nature Research policies, see our Editorial Policies and the Editorial Policy Checklist.

Statistics

For all statistical analyses, confirm that the following items are present in the figure legend, table legend, main text, or Methods section.

n/a	Confirmed	
☐	☒	The exact sample size (n) for each experimental group/condition, given as a discrete number and unit of measurement
☐	☒	A statement on whether measurements were taken from distinct samples or whether the same sample was measured repeatedly
☒	☐	The statistical test(s) used AND whether they are one- or two-sided<br>*Only common tests should be described solely by name; describe more complex techniques in the Methods section.*
☒	☐	A description of all covariates tested
☒	☐	A description of any assumptions or corrections, such as tests of normality and adjustment for multiple comparisons
☐	☒	A full description of the statistical parameters including central tendency (e.g. means) or other basic estimates (e.g. regression coefficient) AND variation (e.g. standard deviation) or associated estimates of uncertainty (e.g. confidence intervals)
☒	☐	For null hypothesis testing, the test statistic (e.g. F, t, r) with confidence intervals, effect sizes, degrees of freedom and P value noted<br>*Give P values as exact values whenever suitable.*
☒	☐	For Bayesian analysis, information on the choice of priors and Markov chain Monte Carlo settings
☒	☐	For hierarchical and complex designs, identification of the appropriate level for tests and full reporting of outcomes
☒	☐	Estimates of effect sizes (e.g. Cohen's d, Pearson's r), indicating how they were calculated

Our web collection on statistics for biologists contains articles on many of the points above.

Software and code

Policy information about availability of computer code

Data collection	Fluidgm CyTOF imaging mass cytometry software v7.0
Data analysis	Cell segmentation pipeline: https://github.com/BodenmillerGroup/ImcSegmentationPipeline/tree/20191002_IMCWorkshop<br>Ilastik: 1.3.3 (Generation of segmentation probability maps)<br>Cellprofiler: 4.2.1 (Signal and background intensity quantification), 3.1.9 (Single cell segmentation)<br>R: 4.2.0 (Single cell data analysis and generation of plots)<br>Rphenograph: 0.99.1<br>All code used for preprocessing and analysing IMC data is available at https://github.com/BodenmillerGroup/SABER-IMC_publication

For manuscripts utilizing custom algorithms or software that are central to the research but not yet described in published literature, software must be made available to editors and reviewers. We strongly encourage code deposition in a community repository (e.g. GitHub). See the Nature Research guidelines for submitting code & software for further information.

Data

Policy information about availability of data

All manuscripts must include a data availability statement. This statement should provide the following information, where applicable:

- Accession codes, unique identifiers, or web links for publicly available datasets
- A list of figures that have associated raw data
- A description of any restrictions on data availability

Raw and processed IMC data will be available upon publication.

Field-specific reporting

Please select the one below that is the best fit for your research. If you are not sure, read the appropriate sections before making your selection.

☒ Life sciences ☐ Behavioural & social sciences ☐ Ecological, evolutionary & environmental sciences

For a reference copy of the document with all sections, see nature.com/documents/nr-reporting-summary-flat.pdf

Life sciences study design

All studies must disclose on these points even when the disclosure is negative.

Sample size	We used mixed cell pellet from three different cell lines, one tonsil tissue, and three melanoma tissues in this manuscript. No sample size determination was carried out since we were not comparing groups but rather developing and testing a method. Signal amplification was successful across these samples, and therefore sufficiently supported the demonstration of the versatility of our method.
Data exclusions	Fields of view without a significant portion of melanoma and immune cells were excluded from the analysis.
Replication	Figures with quantitative analyses showing error bars (S.E.M.) were carried out in three experimental replicates. Especially, signal amplification was evaluated on 3 FFPE sections of cell pellet to confirm the stability of the amplification.
Randomization	Since we were not conducting a biological study, randomization is not relevant. However, FFPE slides for comparing different amplification groups (e.g. standard IMC, SABERx2) were selected randomly. Three melanoma tissues were selected to demonstrate different inflammation states (Inflamed, Excluded, Mixed).
Blinding	Since we were not conducting a biological study, blinding is not relevant. The three melanoma tissues with different inflammation state were stained and imaged simultaneously.

Reporting for specific materials, systems and methods

We require information from authors about some types of materials, experimental systems and methods used in many studies. Here, indicate whether each material, system or method listed is relevant to your study. If you are not sure if a list item applies to your research, read the appropriate section before selecting a response.

Materials & experimental systems

n/a	Involved in the study	
☐	☒	Antibodies
☐	☒	Eukaryotic cell lines
☒	☐	Palaeontology and archaeology
☒	☐	Animals and other organisms
☐	☒	Human research participants
☒	☐	Clinical data
☒	☐	Dual use research of concern

Methods

n/a	Involved in the study	
☒	☐	ChIP-seq
☒	☐	Flow cytometry
☒	☐	MRI-based neuroimaging

Antibodies

Antibodies used	The list of used antibodies are available in supplementary table 2
Validation	All antibodies were validated by test staining on several tissue types including tonsil, melanoma and cell pellets. For validation, expected spatial distribution and expression profile between markers were assessed on the tissues. Cell type specific expression was assessed on cell pellets.

Eukaryotic cell lines

Policy information about cell lines

Cell line source(s)	ZR-75-1: ATCC CRL-1500<br>Ramos: ATCC CRL-1596<br>Jurkat cells: ATCC TIB-152
Authentication	Cell lines were derived from ATCC vials but were not further authenticated
Mycoplasma contamination	Cell lines were derived from ATCC vials but were not further tested

April 2020

Commonly misidentified lines<br>(See ICLAC register)	No commonly misidentified lines were used

Human research participants

Policy information about studies involving human research participants

Population characteristics	Three melanoma tissues had different immune inflammation state (Inflamed, Excluded, Mixed).
Recruitment	Melanoma tissues were only used for demonstrating the detection of immune cell population with T cell co-receptor and ligands by using SABER-IMC.
Ethics oversight	Samples were received from University Hospital Zurich, which was approved by the Ethikkommission Kanton Zürich (KEK-ZH-Nr 2014-0425)

Note that full information on the approval of the study protocol must also be provided in the manuscript.
