# Supplementary material for: DNA-barcoded signal amplification for imaging mass cytometry enables sensitive and highly multiplexed tissue imaging
Source: Nat Methods. 2023 Aug 31;20(9):1304–9. doi: 10.1038/s41592-023-01976-y (PMC10482679; doi:10.1038/s41592-023-01976-y)

Extended Data Fig. 1b (left)

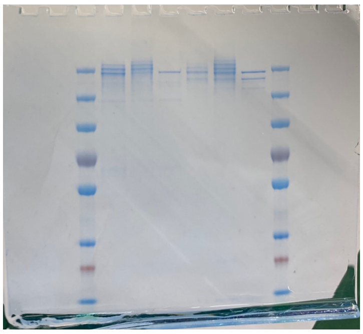

Source gel image

Cropped area

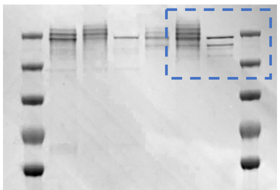

Extended Data Fig. 1b (right)

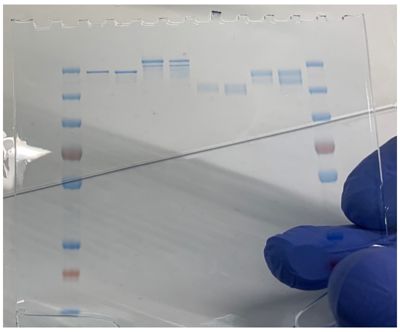

Source gel image

Cropped area

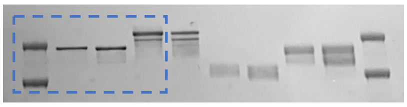

Extended Data Fig. 1c

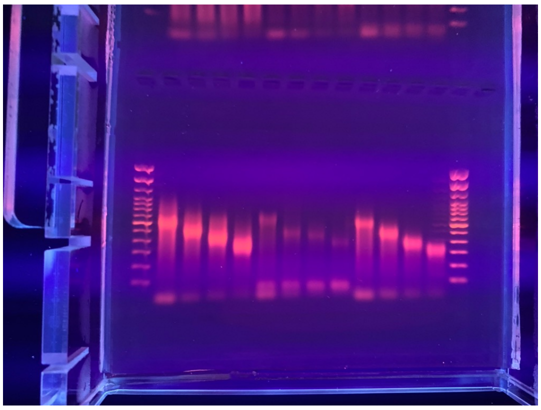

Source gel image

Cropped area

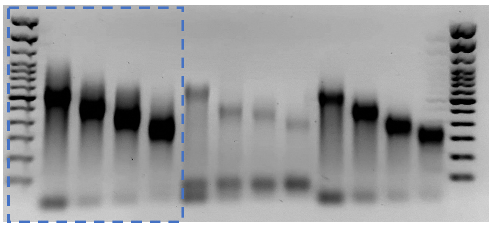

Extended Data Fig. 1e

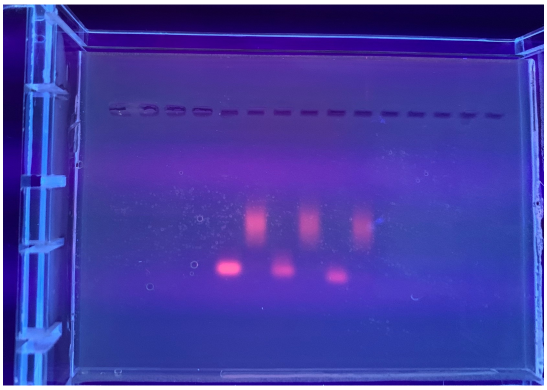

Source gel image

Cropped area

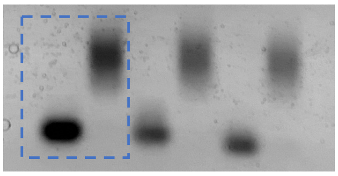

Supplement: Source Data Extended Data Fig. 1 — Unprocessed gel images. [file 41592_2023_1976_MOESM3_ESM.pdf]
